# Detecting COVID-19 infection status from chest X-ray and CT scan *via* single transfer learning-driven approach

**DOI:** 10.3389/fgene.2022.980338

**Published:** 2022-09-21

**Authors:** Partho Ghose, Muhaddid Alavi, Mehnaz Tabassum, Md. Ashraf Uddin, Milon Biswas, Kawsher Mahbub, Loveleen Gaur, Saurav Mallik, Zhongming Zhao

**Affiliations:** ^1^ Department of Computer Science and Engineering, Bangladesh University of Business and Technology, Dhaka, Bangladesh; ^2^ Department of Computer Science and Engineering, Jagannath University, Dhaka, Bangladesh; ^3^ Center for Health Informatics, Macquarie University, Sydney, NSW, Australia; ^4^ Amity International Business School, Amity University, Noida, India; ^5^ Department of Environmental Health, Harvard T H Chan School of Public Health, Boston, MA, United States; ^6^ Human Genetics Center, School of Public Health, The University of Texas Health Science Center at Houston, Houston, TX, United States

**Keywords:** COVID-19, x-ray, CT scan, deep learning, transfer learning, classification

## Abstract

COVID-19 has caused over 528 million infected cases and over 6.25 million deaths since its outbreak in 2019. The uncontrolled transmission of the SARS-CoV-2 virus has caused human suffering and the death of uncountable people. Despite the continuous effort by the researchers and laboratories, it has been difficult to develop reliable efficient and stable vaccines to fight against the rapidly evolving virus strains. Therefore, effectively preventing the transmission in the community and globally has remained an urgent task since its outbreak. To avoid the rapid spread of infection, we first need to identify the infected individuals and isolate them. Therefore, screening computed tomography (CT scan) and X-ray can better separate the COVID-19 infected patients from others. However, one of the main challenges is to accurately identify infection from a medical image. Even experienced radiologists often have failed to do it accurately. On the other hand, deep learning algorithms can tackle this task much easier, faster, and more accurately. In this research, we adopt the transfer learning method to identify the COVID-19 patients from normal individuals when there is an inadequacy of medical image data to save time by generating reliable results promptly. Furthermore, our model can perform both X-rays and CT scan. The experimental results found that the introduced model can achieve 99.59% accuracy for X-rays and 99.95% for CT scan images. In summary, the proposed method can effectively identify COVID-19 infected patients, could be a great way which will help to classify COVID-19 patients quickly and prevent the viral transmission in the community.

## 1 Introduction

COVID-19 disease has caused one of the major pandemic events in human history since COVID-19 cases were first identified in late 2019. This virus, SARS-CoV-2, was initially discovered in Wuhan, China, in November 2019. The world has experienced the outbreak of this virus quickly and it has been not under control due to new viral strains being emerged. The World Health Organization (WHO) declared it a global epidemic due to its fast spread among humans Organization (2020).

COVID-19 has been defined as a respiratory disease because it causes myofascial pain syndrome, sore throat, headache, fever, breathing difficulty, dry cough, and chest infection Huang et al. (2020). In addition, an infected individual might display full symptoms in around 14 days. Over 528 million COVID-19 cases have been recorded in over 200 countries and territories in May 2022, resulting over 6.25 million fatalities Organization et al. (2020). Consequently, the global community currently faces a severe public health threat. The WHO labeled the expansion of disease as a public health crisis of international concern (PHEIC) on January 30, 2020, and acknowledged it as a pandemic on March 11, 2020 Organization (27 April 2020). Researchers have been continuing research to invent an effective vaccine to contain the virus Shah et al. (2021); Ahuja et al. (2021). Several vaccines, including Pfizer-BioNTech and Moderna, have been quickly designed and proven effective. However, many people from developing countries still cannot be vaccinated due to their poor economy and a lack of modern technology to preserve the vaccine. Furthermore, the virus can mutate quickly and infect people with a new strain. The vaccine for tackling a strain has not been fully effective for containing a novel strain. Therefore, we need to identify the infected individuals and isolate them to stop the rapid spread of this virus. However, the identification is very challenging as people infected with this virus show similar pneumonia, fever, and flu symptoms. Although at present, the most reliable identifying process is reverse transcription-polymerase chain reaction (RT-PCR), this process is time-consuming and costly, and the testing kits are limited in storage Wang et al. (2020). To address this issue, researchers have aimed to develop an effective RT-PCR alternative approach to detect viral sequence from possible COVID-19 patients and thus control the virus’s spread.

COVID-19 primarily infects the lungs, and visually marking the affected area aids in rapidly screening infected individuals Chung et al. (2020). Chest radiography images (chest X-Ray or computed tomography (CT) scan) are extensively used as a visual signal of lung Chung et al. (2020). So Screening with chest X-rays and CT scan is a potential approach to detecting COVID-19 afflicted patients. Deep learning (DL) approaches primarily focus on automatically collecting features from images and classifying them. DL applications are successfully implemented in the classification of medical images and clinical decision-making tasks Greenspan et al. (2016). The use of machine learning (ML) algorithms and software, or artificial intelligence (AI)Gaur et al. (2022); Biswas et al. (2021), to replicate human cognition in the analysis, display, and comprehension of complicated medical and health-care data is referred to as AI in healthcare Biswas Milon and Kawsher. (2022). Deep transfer learning also is being successfully applied in many medical sectors. For example PreRBP-TL is used for the reconstruction of gene regulatory networks, modeling of gene expression from single-cell data, and prediction of genomic properties such as accessible regions, chromatin connections, and TFBSs. Furthermore, deep learning techniques in bioinformatics offer a variety of uses, as well as tools and methodologies to quickly unfold the hidden information inside the DNA sequences‘Biswas Milon. (2022). In COVID-19 research, experts work round the clock to develop a reliable framework to diagnose COVID-19 utilizing medical imagery like lungs X-rays imaging and CT scan carrying deep learning(DL) technologies. And, numerous studies also offer an excellent diagnostic technique based on a DL algorithm for the recognition of COVID-19 patients Loey et al. (2020); Horry et al. (2020); Lahsaini et al. (2021); Maghded et al. (2020); Apostolopoulos and Mpesiana (2020); Ghose et al. (2021); Nayak et al. (2021); Pham (2021). Lahsaini et al. (2021) introduced a transfer learning-based system based on DenseNet201 with a visual explanation. They achieved an accuracy of 98.8% for identifying COVID-19 patients. At the same time, Maghded et al. (2020) developed a smartphone-based architecture to identify COVID-19 considering mass people. However, the accuracy and reliability of this approach are not acceptable. Apostolopoulos and Mpesiana (2020) introduced a MobileNetv2 model based on transfer learning for self-acting COVID-19 identification. The study used 1427 X-Rays and acquired 98.66% sensitivity, 96.78% accuracy, and specificity of 96.46%. In another study, Ghose et al. (2021) proposed a novel Convolutional neural Network (CNN) based model to identify COVID-19 individuals with an accuracy of 96%. For this research purpose, Panwar et al. (2020a) used Chest X-ray and CT scan images with a DL strategy for color viewing and quick COVID-19 data identification. On the other hand, Panwar et al. (2020b) utilized the nCOVnet base approach in X-ray images to find privacy risks inability to detect the COVID-19 instance. Furthermore, they demonstrated that the proposed technology is faster than a standard RT-PCR testing kit and can therefore differentiate COVID-19 from other respiratory disorders like Pneumonia. Realizing the effectiveness of this technology Luján-García et al. (2020) used simple DL models with X-rays as input and created 36 convolution neural layers to reach a precision score of 0.843 for identifying Pneumonia. To improve accuracy, a strategy was devised by Bhattacharyya et al. (2022) to recognize COVID-19 patients from X-ray images efficiently. First, they merged DL-based image segmentation and classification models to improve results. Finally, they categorized the convoluted features by applying ML methods, including SoftMax, RF, SVM, and XG Boost. The VGG 19 architecture blended with the BRISK key-points mining strategy with RF as the categorization layer got a significant accuracy of 96.6%. Loey et al. (2020) suggested a Deep Transfer Learning-based method for identifying COVID-19 using CT scan images of the chest. To make this process more faster and more automated, Alshazly et al. (2021) exhibited a deep learning model train for chest CT scan. For the first time, Maghded et al. (2020) stated an IoT based strategy for recognizing COVID-19, and they could establish a strategy for diagnosing COVID-19 using built-in sensors with CT scan images. They further claimed that their malware detection solution for smartphones was not only the most cost-effective and user-friendly but also the fastest. Their approach, however, has a significant flaw in that they do not address the accuracy of COVID-19 recognition. Furthermore, the individuals who take advantage of a smartphone’s standard sensors may pose security issues being their health conditional data public, which is not desirable. Unfortunately, they did not devote any attention to finding a solution to this problem. On the other side, somebody tries to make those detection more reliable by joining multiple architectures. For example, Aslan et al. (2021) effectively merged two pre-trained Alexnet architectures (transfer learning and BiLSTM layer) for COVID-19 separation, claiming that the stated hybrid system could do the better COVID-19 recognition than almost any single model base architecture. Furthermore, Ter-Sarkisov (2022) presented a COVID-CT-MaskNeT model to forecast COVID-19 in chest CT scan images.

Upon 21,192 test images, they obtained 90.80% sensitivity for COVID-19 cases, 91.62% sensitivity for Pneumonia cases, a mean accuracy of 91.66% and an F1-score of 91.50% by training only a tiny proportion of the model’s parameters.

As reviewed above, approaches like CNN, DL, and transfer learning have all been employed to diagnose COVID-19. AI technology can introduce a new era in medical science by allowing for rapid illness detection and categorization, perhaps step-down the transmission of the COVID-19 virus. Representative works are briefly summarized in Table 1.

**TABLE 1:** Studies evaluating DL methods for COVID-19 identification.

Study	Sample size and type	Models	Accuracy (%)
Loey et al. (2020)	760 CT scan	CGAN, AlexNet, GoogleNet, VGGNet16, VGGNet19, ResNet50	82.9
Alshazly et al. (2021)	2,482 CT scan	SqueezeNet, ResNet50, InceptionV3, ResNet101, ResNeXt50, ResNeXt101, Xception, DenseNet169, DenseNet201	93.7
Maghded et al. (2020)	Real-time CT scan images	Smartphone, onboard sensors, ML models	N/A
Lahsaini et al. (2021)	4,986 CT scan	DenseNet121, DenseNet201, VGG16, VGG19	98.8
Panwar et al. (2020b)	337 X-Rays	nCOVnet	98.97
Aslan et al. (2021)	2,905 X-Rays	mAlexNet– BiLSTM (Hybrid) architecture	98.70
Purohit et al. (2022)	5,220 CT scan	Deep Learning	96.47
Bassi and Attux (2022)	2,064 X-Rays	Deep CNN	99.02
Mahbub et al. (2022)	1,200 X-Rays	Customized DNN	99.87
Mahbub et al. (2021)	2,541 X-Rays	RegNet Structured DL	99.02
Gaur et al. (2021)	3,106 X-Rays	EfficientNetB0, VGG16, InceptionV3	92.93

We propose an enhanced DL architecture to recognize COVID-19 and normal patients from X-Rays and CT scan utilizing the transfer learning strategy based on a DenseNet169 model. The significant contributions are:

• We have proposed a customized deep network to assist in the fast diagnosis of COVID-19 patients precisely by using a single transfer learning technique.
• An in-depth experimental analysis is carried out in view of accuracy, precision, recall, F1-score, and confusion matrix to evaluate the suggested model’s performance. Utilizing our proposed model, we have identified CT scan images with an accuracy rate of 99.95% for two classes (normal and COVID-19 patients). Similarly, for X-rays, our proposed model has shown a remarkable performance with a detection accuracy of 99.59%.

## 2 Materials and methods

**FIGURE 1:**
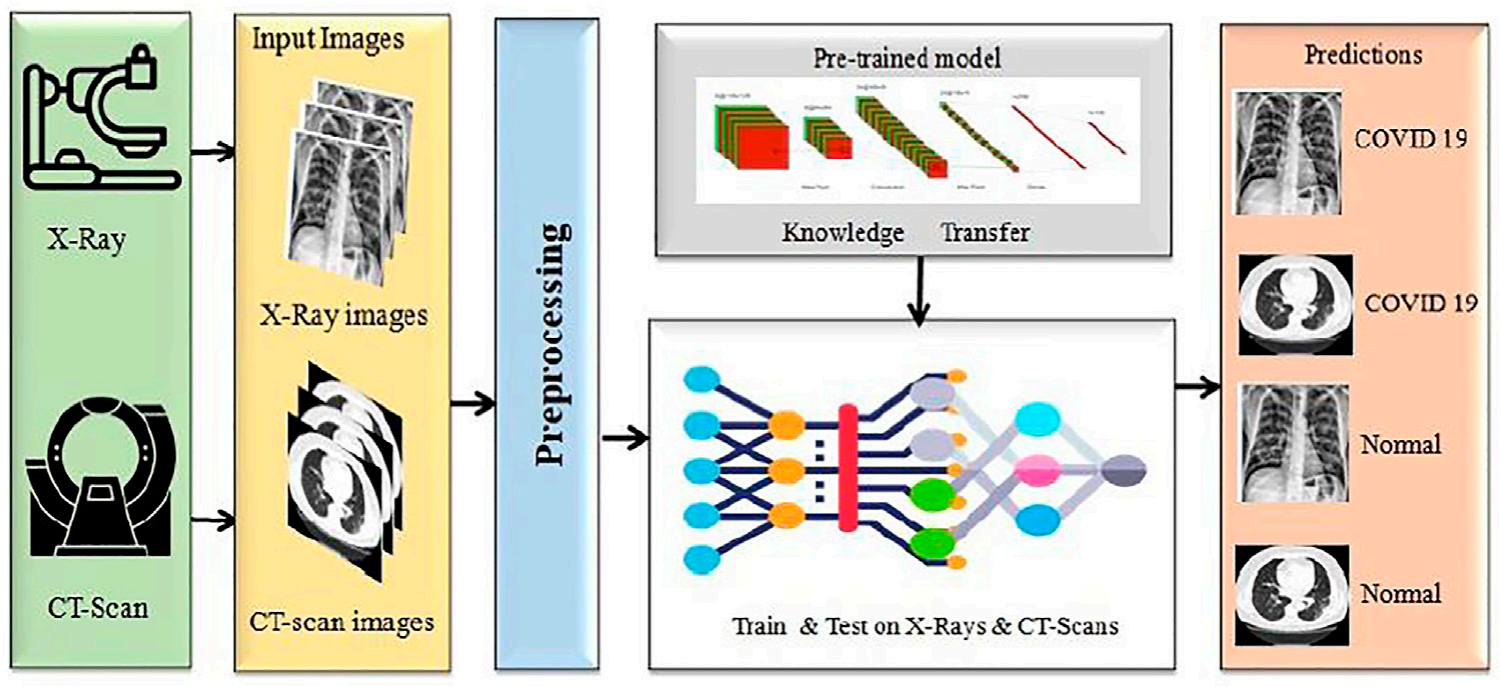
The proposed COVID-19 detection framework using both X-ray and CT-scan.

We proposed a model to distinguish COVID-19 patients from non-COVID-19 through chest X-rays and CT scan using a DL network based on the DenseNet169 introduced by Huang et al. (2017). This task uses transfer learning techniques, typically utilized in applications with a small data set, and retrains a pre-trained model on a massive dataset like ImageNET. Our paper uses a single transfer learning approach to reduce computational complexity. It can improve the model’s total training time and allow the deployment of a smaller dataset with a more complex architecture. Before training the model, we did some significant pre-processing on the training data. The block diagram of this experiment is presented in Figure 1.

### 2.1 Materials

We proposed a DL based model aimed in identifying COVID-19 patients utilizing X-rays and CT scan data gathered from Siddhartha and Santra (2020) and ARIA (2021). The tests are carried out on a local machine with an i5-8265U processor, 8GB RAM, and Google-Collab for GPU. The transfer learning technique is applied to CNN models in this study, frequently utilized in applications with restricted data sets.

#### 2.1.1 Dataset collection

##### CT scan images for COVID-19 and normal individuals

**FIGURE 2:**
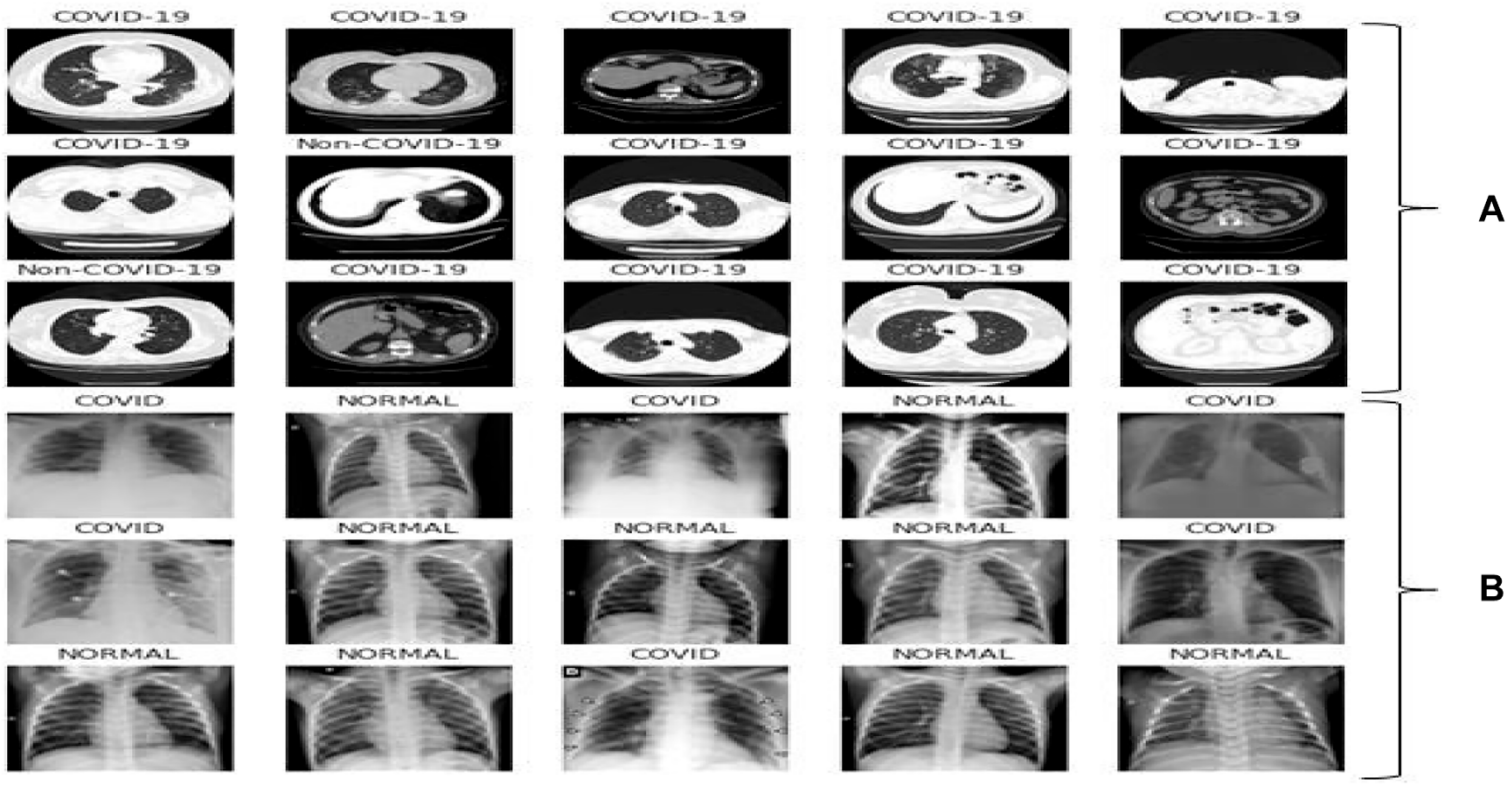
Representative samples: **(A)** CT scan samples from the dataset ARIA (2021), **(B)** X-ray samples from the dataset Siddhartha and Santra (2020).

This dataset carries 8439 CT scan, of which 7495 are the COVID-19 belonging to 190 people and 944 CT-Sans of 59 peoples without COVID-19, pneumonia patients, and otherwise healthy individuals. This daraset was generated from ARIA (2021). The disease status of the suspected patients in this group was confirmed using an RT-PCR test. Figure 2A showed some representative CT scan of this dataset associated with normal and COVID-19 individuals.

##### X-rays for COVID-19 and normal individuals

The second dataset contains 1626 images for COVID-19 patients and 1802 images for normal individuals found in Siddhartha and Santra (2020). The disease status of the suspected patients was confirmed using an RT-PCR test and is annotated by radiologist. Figure 2B showed few X-ray images associated with the dataset.

### 2.2 Methods

The research aims to determine the optimal architecture for classifying individuals with COVID-19 positive or normal. We have chosen a few CNN architectures that have produced outstanding results on the ImageNET and CiFAR datasets for the said purpose.

#### 2.2.1 Pre-processing

According to the research, the effectiveness of medical imaging computer vision tasks in DL is not mainly attributable to CNN models; instead, image pre-processing plays a significant role. As the primary step of pre-processing, data normalization is executed to preserve the quality of the images, which is critical in the analysis of X-ray and CT scan images according to Patro (2015). First, we computed the pixel-level global average (SD) values for all of the images and afterward normalized the data with the formula below in Eq. 1:

$$X_{i}=\frac{X_{i}-\bar{x}}{\epsilon +\sigma }$$ (1)

where *σ* is the SD, $\bar{x}$ is the global average of the data set X, and *ϵ* = 1 × 10^–10^ is an influential value to control the denominator of being 0.After that normalization, the images were standardized by converting the pixel values of each image (0.255) and then (0,1) by obtaining an integrated scale for DL model input, as the images would be standardized during training. Even though CNNs rely heavily on a dataset to improve their effectiveness and protect model over-fitting in accordance to Shorten and Khoshgoftaar (2019); we employed the data augmentation procedure to quickly enhance the dataset, which has a considerable effect on the dataset’s quality and shape and the capabilities of the model during training. At this point, we have deployed data augmentation strategies to enhance the dataset automatically. In our case, the data augmentation tool first re-sampled the images (reducing or enlarging during the augmentation process). A randomized repetition is then applied. Then, we used a height change to flip the images horizontally. Again, the zooming range is set to 30%, and finally, we make a random contrast of 0.20. Input image dimension has been reformed to 224 × 224 × 3, although this method can be applied to any size in any image. Then, in the study, the dataset was randomly partitioned into two parts: 20% for testing and the remaining 80% for training, and then 20% of the training set is used for validation.

#### 2.2.2 DenseNet169

**FIGURE 3:**
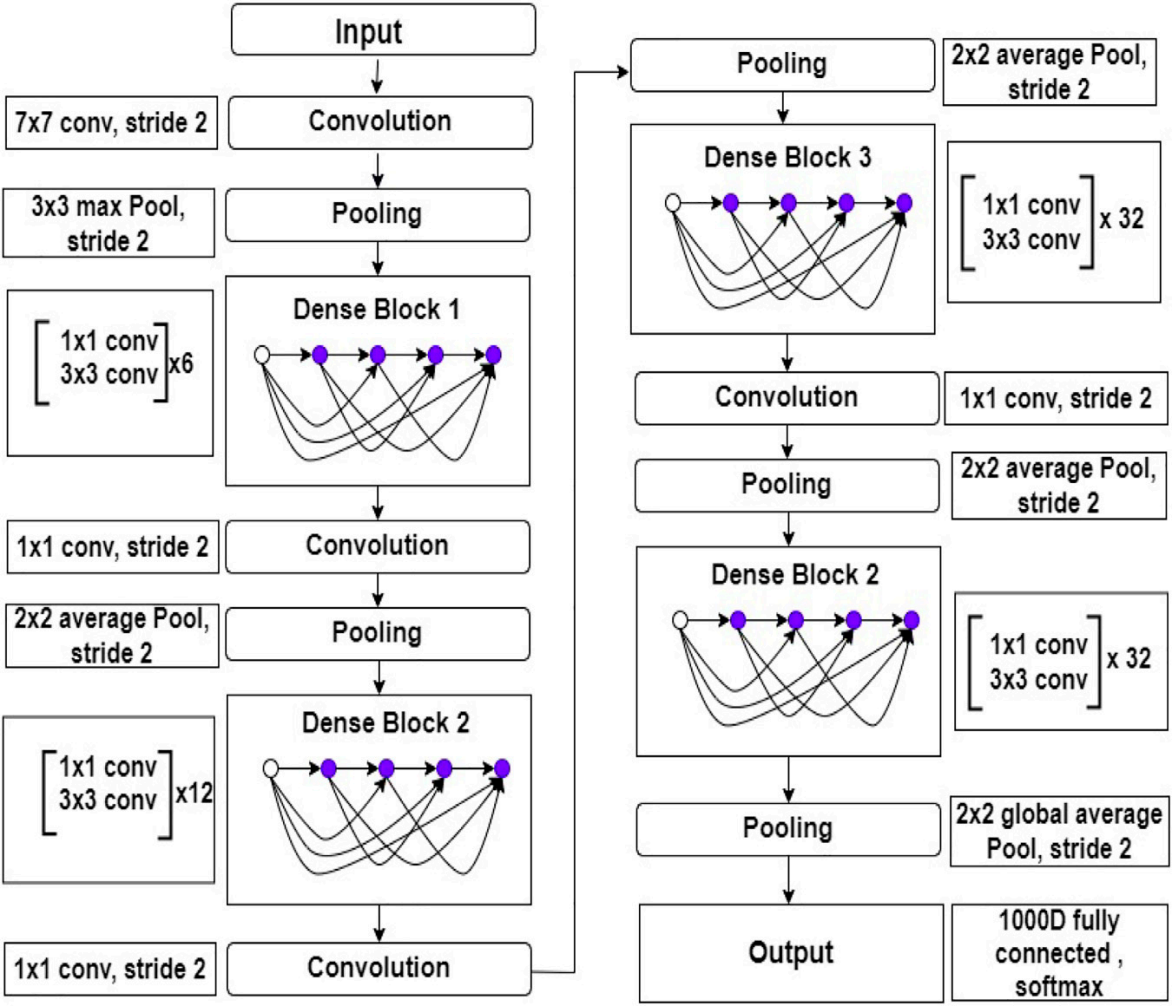
Inside view of DenseNet169 deep learning model. This figure shows the layers architecture with layers number.

In limited data classification tasks, transfer learning has proven to be reliable. Therefore, tuning on Deep transfer learning (DTL) can help us get better results. We suggested the DenseNet169 model, which uses transfer learning to draw out features simultaneously and utilize their weights learned on the ImageNET dataset to reduce computation time. Huang et al. (2017) presented this type of model for the first time in 2017, and it contains one convolution and pooling layer at the start, three transition layers, four dense blocks, and a classification layer is used after that. For example, with stride 2, the first convolutional layer conducts 77 convolutions, followed by a max-pooling of 33 with stride 2. After that, the network consists of a dense block, followed by three sets consisting of a transition layer and a dense block. Huang et al. (2017) made dense connectivity by bringing indirect connections from any layer to any other layer in the network. As a result, the network’s *l* ^*t*^ *h* layer collects the characteristics maps of all the preceding layers, improving gradient flow throughout the whole network. The DenseNets architecture is separated into the several densely linked dense blocks indicated above because convolutional neural networks are primarily designed to diminish the size of feature maps. The layers that appear between these large blocks are known as transition layers. A batch normalization layer, an 11 convolutional layer, and a 22 average pooling layer with a stride of 2 make up the network’s transition layers. The detailed architecture of DenseNet169 is shown in Figure 3.

#### 2.2.3 Proposed method

We proposed a customized DenseNet169-based model for COVID-19 recognition. The feature extraction layers were kept in the pre-trained DenseNet169 and then added a convolution layer, the Global Average Pooling layer, the batch normalization layer. We apply two dense layers with 512 and 256 neurons following batch normalization, and a 20% dropout layer is utilized in front of the second thick layer to avoid overfitting. The final classification or output layer has two neurons and employs the SoftMax function, which connects all neurons in almost the duplicate layer to the next layer. We have two outputs in our study. We employed SoftMax to categorize them into two groups: one for not infected or normal patients and another for infected or COVID-19 positive patients using Eq. 2.

$$\begin{array}{c} \varphi \left(\chi_{i}\right)=\frac{\exp\left(\chi_{i}\right)}{\sum_{j=0}^{k}\exp\left(\chi_{j}\right)} \end{array}$$ (2)

where *χ* is input value and *k* implies the number of classes to be forecast.

**FIGURE 4:**
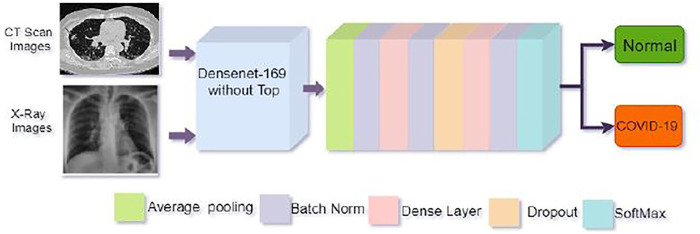
Architecture of the proposed model. The top of the pre-trained DenseNet169 model was removed and then added some layers as shown in the figure for better COVID-19 detection.

The strategy applied in this study has been depicted in Figure 4.

#### 2.2.4 Feature extraction procedure

Throughout the feature extraction phase, convolutional neural layers is used.Batch normalization is utilized to avoid model overfitting, weight regularization, and dropout approaches. The weights are regularized using the Euclidean norm (L2), with coefficient values ranging from (0.001–0.01) and a 20% weight dropout. The early stages of the pre-trained DenseNet169 network, which has a scalable architecture for image classification, are employed for enhanced features extraction influenced by the transfer learning technique. DenseNet169s convolutional cells are used in Figure 4 with their structure.The feature extraction blocks are avoided and have non-trainable parameters throughout the training mode, and Adam is used as the optimizer. We have used the cost-sensitive binary cross-entropy loss function presented in equation 3

$$I\left(y,\varphi \right)=\;-\omega_{k}\left(y_{k}log\left(\varphi_{k}\right)+\left(1-y_{k}\right)\log\left(1-\varphi_{k}\right)\right)$$ (3)

Where, *k* is the number of class (in this work the classes are COVID-19 and normal), and *φ*_*k*_ is the SoftMax function or anticipated probability of class *k*, *y* is binary indicator (0 or 1) and *ω*_*k*_ is weight for class *k*.

We set the initial learning rate 1 × 10^–6^. If no further improvement in the accuracy and the validation loss remained steady, the learning rate would be cut by 20% to a minimum of 1 × 10^–6^ for every ten epochs. The training process would end if the validation loss did not change for at least 15 epochs, and the best weights would be determined. For the batch size, 32 is selected. The number of epochs evaluated is 50.

## 3 Results and discussion

### 3.1 Performance evaluation matrices

When evaluating classification models, the most frequently used metrics are Accuracy, Precision, Recall, and F-1 Score. The formulae for the matrices are as follows:

$$\begin{array}{c} Accuracy\;\left(ACC\right)=\frac{\left(TP+TN\right)}{\left(TN+FP+TP+FN\right)} \end{array}$$ (4)

$$\begin{array}{c} Precision\;or\;Positive\;Predictive\;Value\;\left(PPV\right)=\frac{TP}{\left(TP+FP\right)} \end{array}$$ (5)

$$\begin{array}{c} Sensitivity\;\left(SEN\right)=\frac{TP}{\left(TP+FN\right)} \end{array}$$ (6)

$$\begin{array}{c} F1-score=\frac{2*SEN*PPV}{SEN+PPV} \end{array}$$ (7)

$$\begin{array}{c} False\;positive\;rate\left(FPR\right)=\frac{FP}{\left(FP+TN\right)} \end{array}$$ (8)

$$\begin{array}{c} False\;negative\;rate\left(FNR\right)=\frac{FN}{\left(TP+FN\right)} \end{array}$$ (9)

where TP, FP, TN and FN denote true positive, false positive, true negative and false negative values respectively.

### 3.2 Experimental results

We gathered the datasets from ARIA (2021) and Siddhartha and Santra (2020). Then we performed some pre-processing such that all of the photos are 224 × 224 pixels on both dataset. Next, we employed data augmentation strategies to develop generalization by combining several strategies.

#### Results for CT scan images

**FIGURE 5:**
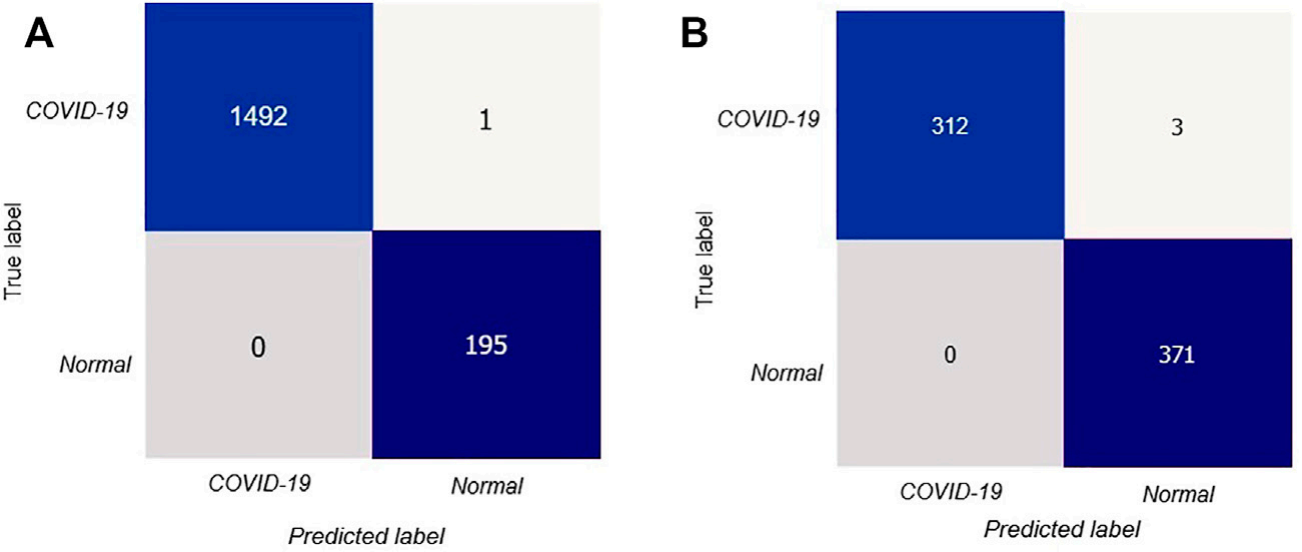
Confusion matrix for the proposed model: **(A)** confusion matrix on CT scan, **(B)** confusion matrix on X-rays.

After that, we have created a confusion matrix to calculate the performance of our suggested design. Figure 5A depicts the confusion matrix of the stated model for test situations on CT scan images.

The confusion matrix shows that out of 1688 test photos, only one image was detected incorrectly by the stated model with better and consistent true negative and true positive values. Thus our proposed deep learning technique can precisely classify COVID-19 patients.

**FIGURE 6:**
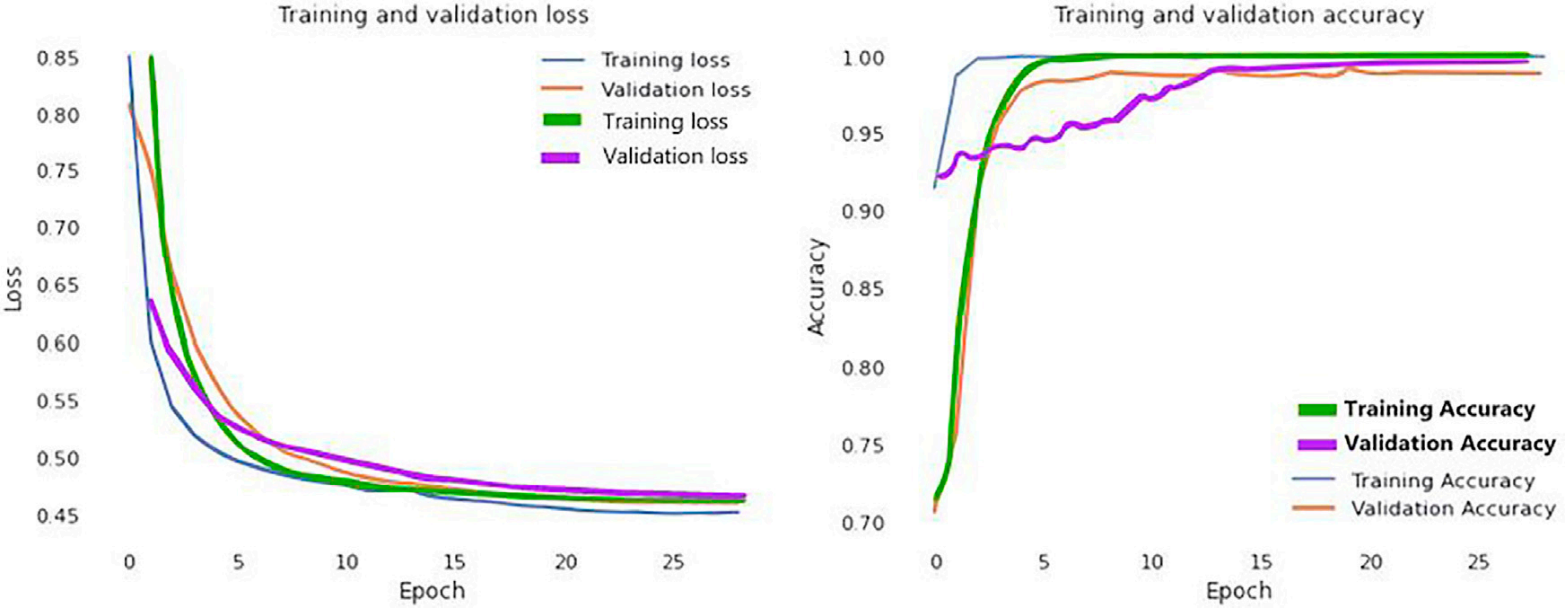
Loss and accuracy curves of the proposed model (Blue and yellow curves represents for CT scan images; green and purple curves ensembles for the X-Ray images).

In addition, Figure 6 visually examines the efficiency of the introduced DL model in the learning and validation steps to comprehend the accuracy and loss (blue and yellow color curves). The accuracy of our suggested technique rose by 92% after the 10th epoch, which indicates that the presented methodology could be deployed to detect COVID-19 quickly. Correspondingly, training and validation accuracies are 99.98 and 99.79% at epoch 25. Furthermore, the proposed method provides that learning and validation losses are 0.12 and 0.15%, accordingly.

Table 2 summarizes the ACC, SEN, PPV, and F1-score for each class referred to. For COVID-19 cases, the suggested DenseNet169 network has 99.96% ACC, 99.93% SEN, 99.98% F1-score, and 100% PPV. In normal situations, an ACC of 99.94%, PPV of 99.48%, SEN of 100%, and F1-score of 99.99% were obtained.

**TABLE 2:** Experimental results obtained by the proposed model for COVID-19 and normal cases on CT scan.

Class	Performance (%)			
	ACC	PPV	SEN	F1-score
COVID-19	99.96	100	99.93	99.98
Normal	99.94	99.48	100	99.99

**FIGURE 7:**
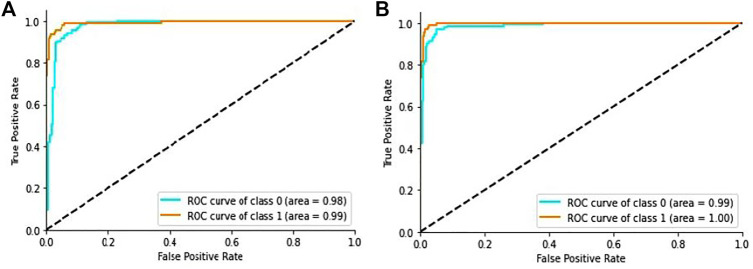
ROC curve of the proposed model: **(A)** ROC curve on CT scan images, **(B)** ROC curve on X-ray images.

In addition, the ROC curves between the actual positive rate and the false positive rate were introduced to see the general evaluation, as shown in Figure 7A. The region under the ROC curve (AUC) was 99.9% for the proposed DL method.

In this study, our suggested model has been compared to four different DL models: VGG16, VGG19, Xception, and Inception-V2-Resnet. Furthermore, we trained all architectures using 32-bit batches for 25 epochs with the same dataset ARIA (2021). Finally, the outcomes are compared using the assessment measures indicated before.

The obtained performances of various models are shown in Table 3, where the introduced DenseNet169 model outperforms other architectures in terms of ACC, PPV, SEN, and F1-score. Furthermore, DenseNet169 has the best SEN of 100%, which is beneficial in medical diagnostic aid because this metric is vital in general and determination support for COVID-19. High sensitivity is, in fact, synonymous with a low false negative rate. False negatives are people afflicted by COVID-19 but diagnosed COVID-19 negative by the model. Such an error can result in the patient’s death. As demonstrated in Table 3, our model also produces good performance in all the other terms.

**TABLE 3:** Experimental results obtained with proposed model and different DL models on CT scan images.

Classifier	Performance (%)			
	ACC	PPV	SEN	F1-score
VGG16	95.69	97.25	95.70	96.70
VGG19	94.20	93.70	96.40	94.55
Xception	96.70	98.40	96.20	97.50
Inception-V2-Resnet	96.90	96.64	97.20	96.50
Densenet169	98.90	99.04	97.98	98.84
Proposed work	99.95	99.74	99.97	98.99

#### Results for X-Ray images

In Figure 5B we showed the confusion matrix generated on X-ray images data Siddhartha and Santra (2020) to determine the performances of proposed model.

The confusion matrix symbolized that our proposed DL model performed very well in precisely classifying COVID-19 patients.

We further visualized the accuracy and loss curve for training and validation in Figure 6 to show the achievement of the presented CNN model. These accuracy and loss curves (green and purple color curves) clearly suggested that the proposed model did well without overfitting and underfitting. For X-rays dataset, we set the epoch number 25 instead as well. After 25 epochs, we found training and validation accuracies 99.96 and 99.83% respectively.

In Table 4, we summarized the results obtained by applying the dataset Siddhartha and Santra (2020). Here, we also considered the evaluation metrics in term of ACC, SEN, PPV, and F1-score for each class. On an average, the proposed model achieved 99.59% ACC, 99.50% SEN, 100% F1-score, and 99.50% PPV on X-ray images.

**TABLE 4:** Experimental results obtained by the proposed model for COVID-19 and normal cases on X-rays.

Class	Performance (%)			
	ACC	PPV	SEN	F1-score
COVID-19	99.68	100	99.00	100
Normal	99.49	99.00	100	100

Additionally, we generated the ROC curves between the actual positive rate and the false-positive-rate with a view to determining the general achievement shown in Figure 7B. We obtained the Area Under the ROC curve (AUC) 99.2% for the proposed architecture.

In this study, our suggested model has been compared to four different architectures:Keeping the parameter same, we also trained VGG16, VGG19, Xception, and Inception-V2-Resnet on X-rays dataset Siddhartha and Santra (2020) to compare the outcomes with our model.

Table 5 depicted the comparative performances of these model with our developed model taking the evaluation criteria ACC, PPV, SEN, and F1-score. As demonstrated in Table 5, the suggested model also produced good performance in all the evaluation metrics with an accuracy of 99.56% on chest X-ray images.

**TABLE 5:** Experimental results obtained with proposed model and different DL models on X-ray images.

Classifier	Performance (%)			
	ACC	PPV	SEN	F1-score
VGG16	94.69	93.59	96.6	94.72
VGG19	92.2	91.7	97.4	93.55
Xception	95.66	96.89	94.5	96.68
Inception-V2-Resnet	96.78	96.44	96.2	94.5
Densenet169	97.8	97.04	96.98	95.84
Proposed work	99.59	99.50	99.50	100

**FIGURE 8:**
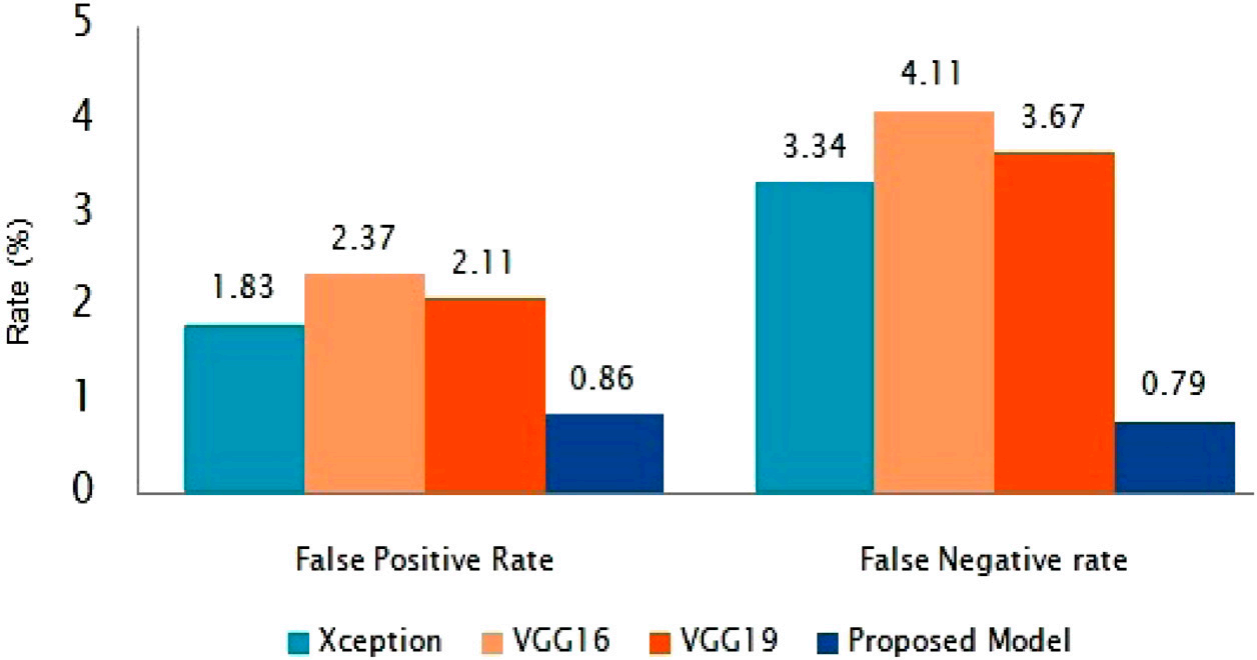
FPR and FNR bar graphs for the proposed model with different models.

The suggested classification performance has been further examined using average false positive rate and false negative rate bar diagrams, as shown in Figure 8. The FPR and FNR values should have been much smaller for improved classifier performance. Our presented method gains the smallest FPR and FNR, which are 0.85 and 0.79%, respectively, as shown in Figure 8.

### 3.3 Comparison with existing methods

As demonstrated in Table 6 and Table 7, we assessed our model to other state-of-the-art DL techniques for recognizing COVID-19 on CXR images and chest CT scan images. For the categorization of COVID-19 and other individual’s images in our dataset, all of the algorithms in Tables 6, 7 was re-implemented. To identify COVID-19, Bhattacharyya et al. (2022) employed VGG19 to extract semantic features with BRISK and used RF as a classifier in the output layer and discovered that the average accuracy, sensitivity, precision, and F1-score was 96.6, 95, 95.0, 95.0%, correspondingly. Researchers Hussain et al. (2021) employed a transfer learning technique adopting MobileNet-v2 as a base model and obtained average classification accuracy, positive predictive value, and sensitivity of 99.12, 99.27, and 97.36%, respectively. Applying the transfer learning technique-based on ResNet-34 model, Nayak et al. (2021) obtained the accuracy, sensitivity, and precision of 95.29, 92.97, and 96.46%. Correspondingly, the average classification accuracy, sensitivity, and F1-score of a tuned AlexNet model were 95.72, 93.59, and 96.78% which was described by Pham (2021). Rahman et al. (2021) had proposed a novel DL model and obtained 99.65% accuracy, 100% precision, 95.59% recall, and 94.56% F1-score. Table 6 proved that our suggested technique outstrips the recently developed COVID-19 identification techniques on chest X-rays.Besides the X-rays, CT scan images are also being used as a meaningful resources for COVID-19 detection. Using CT scan, Ter-Sarkisov (2022) designed a COVID-CT-MAsk-Net model and gained 90.80% sensitivity for COVID-19 cases, 91.62% sensitivity for Pneumonia cases, and on an mean accuracy of 91.66%, and an F1-score of 91.50%. In another work, Purohit et al. (2022) utilized 5,220 CT scan to differentiate COVID-19 from normal where 2,760 images were on COVID-19 patients. Their model able to gained 96.47% ACC, 96% PPV and 96% F1-score. Research works done by Kogilavani et al. (2022); Kundu et al. (2022); Abraham and Nair (2022); Basu et al. (2022); Islam and Nahiduzzaman (2022); Shaik and Cherukuri (2022) applied the same datasets containing 2,482 CT scan where 1,252 CT scan were COVID-19 infected patients. All of them achieved around 98–98.99% which was good. From the Table 7, we find that Zhou et al. (2021) gained 99.05% ACC, 99.05% SEN and 98.59% F1-score which are very close to our proposed model.After summarised in Tables 6, 7, we have found that our proposed model can differentiate COVID-19 and normal patients efficiently. Thus, it is worth to claim that the introduced model is comparable with the recent published works and can be used to quick and primary identification of COVID-19 infected persons.

**TABLE 6:** Performance comparison of the stated COVID-19 identification model with others works on X-rays.

Study	Data size	Performance (%)			
		ACC	PPV	SEN	F1-score
Nayak et al. (2021)	500 X-Rays	95.29	96.46	92.97	97.5
Bhattacharyya et al. (2022)	930 X-Rays	96.60	95.00	95.00	95.00
Aradhya et al. (2021)	306 X-Rays	79.76	–	–	–
Pham. (2021)	3314 X-Rays	95.72	94.64	93.59	96.78
Ismael and Şengür. (2021)	380 X-Rays	94.70	97.78	94.00	95.92
Narin et al. (2021)	14,194 X-Rays	96.10	91.8	96.6	83.5
Bassi and Attux. (2022)	2,064 X-Rays	99.02	–	99.82	99.92
Mukherjee et al. (2021a)	336 X-Rays	95.83	97.31	98.21	93.45
Hussain et al. (2021)	7,390 X-Rays	99.12	99.27	97.36	95.36
Rahman et al. (2021)	18,479 X-Rays	99.25	99.00	95.59	94.56
Mukherjee et al. (2021b)	260 X-Rays	96.92	99.22	100.00	94.20
Proposed model	3,428 X-Rays	99.59	99.50	99.50	100.00

**TABLE 7:** Performance comparison of the stated COVID-19 identification model with others works on CT scan.

Study	Data size	Performance (%)			
		ACC	PPV	SEN	F1-score
Purohit et al. (2022)	5,220 CT scan	96.47	96.00	96.73	96.00
Kogilavani et al. (2022)	2,481 CT scan	97.53	97.50	97.50	97.00
Kundu et al. (2022)	2,478 CT scan	97.81	97.77	97.81	97.77
Abraham and Nair (2022)	746 CT scan	91.60	90.40	91.70	91.00
Basu et al. (2022)	2,482 CT scan	98.87	–	–	91.95
Islam and Nahiduzzaman (2022)	2,482 CT scan	99.73	99.46	100	99.73
Shaik and Cherukuri (2022)	2,482 CT scans	98.99	98.98	99.00	98.99
Ter-Sarkisov (2022)	14,194 CT scan	91.66	92.04	90.89	91.50
Zhou et al. (2021)	5,000 CT scan	99.05	99.6	99.05	98.59
Proposed model	8,439 CT scan	99.95	99.74	99.97	98.99

## 4 Conclusion and future direction

This work utilise bench-marking approaches and alternative models to categorize COVID-19 X-ray and CT scan images. For the COVID-19 test, this approach can save RT-PCR screening kits (and cost) and take less time than the RT-PCR test. Our study uses a customized deep network based on DenseNet169 to support the fast diagnosis of COVID-19 infected individuals using a precise transfer learning approach. As a result, the accuracy rate for COVID-19 and normal cases identification is 99.95%. For the sensitiveness and aggressive spreading system of the COVID-19, we also focus on evaluating the proposed method using the F1-score, sensitivity (SEN), and precision (PPV) and have achieved 98.99% F1- score, 99.97% SEN, and 99.74% PPV, respectively for CT scan images. In addition, it is worth mentioning that our method has the lowest FPR and FNR when compared to state-of-the-art algorithms, which are 0.86 and 0.79%, respectively. We also used chest X-ray images to train and test our model and found that our proposed model performed well in chest X-rays with an detection accuracy of 99.59%. The proposed model is more accurate and can quickly detect COVID-19 patients to make them isolated quickly and thus can contribute to stop the spreading of infection. However, we do not use particular parameters for chain-smokers or various drag-affected peasants’ lung images. In the future, we will give special attention and care to classify patients with other lung disease.

## Data Availability

Publicly available datasets were use for method development and analysis in this study. These data can be found here at: CT scan-https://www.kaggle.com/datasets/mehradaria/covid19-lung-ct-scans (2021), and X-Rays-https://data.mendeley.com/datasets/rscbjbr9sj/3.(2020).
